# Thickness-dependent humidity sensing by poly(vinyl alcohol) stabilized Au–Ag and Ag–Au core–shell bimetallic nanomorph resistors

**DOI:** 10.1098/rsos.171986

**Published:** 2018-06-06

**Authors:** Parag Adhyapak, Rohini Aiyer, Sreekantha Reddy Dugasani, Hyeong-U Kim, Chung Kil Song, Ajayan Vinu, Venkatesan Renugopalakrishnan, Sung Ha Park, Taesung Kim, Haiwon Lee, Dinesh Amalnerkar

**Affiliations:** 1 Centre for Materials for Electronics Technology (C-MET), Panchwati, Off Pashan Road, Pune 411008, India; 2 Department of Physics, Savitribai Phule Pune University, Ganeshkhind, Pune 411007, India; 3 Department of Physics, Sungkyunkwan University, Suwon 16419, South Korea; 4 Sungkyunkwan Advanced Institute of Nanotechnology (SAINT), Sungkyunkwan University, Suwon 16419, South Korea; 5 Institute of Nano Science and Technology, Hanyang University, Seoul 04763, South Korea; 6 Global Innovative Center for Advanced Materials, Faculty of Natural Built Environment and Engineering, University of Newcastle, Callaghan, Newcastle, NSW 2308, Australia; 7 Center for Renewable Energy Technologies, Department of Chemistry and Chemical Biology, Northeastern University, Boston, MA 02115, USA; 8 Children's Hospital, Harvard Medical School, Boston, MA 02115, USA

**Keywords:** Ag, Au, core–shell, nanostructures, humidity sensing

## Abstract

We herein report a simple chemical route to prepare Au–Ag and Ag–Au core–shell bimetallic nanostructures by reduction of two kinds of noble metal ions in the presence of a water-soluble polymer such as poly(vinyl alcohol) (PVA). PVA was intentionally chosen as it can play a dual role of a supporting matrix as well as stabilizer. The simultaneous reduction of metal ions leads to an alloy type of structure. Ag(c)–Au(s) core–shell structures display tendency to form prismatic nanostructures in conjunction with nanocubes while Au(c)–Ag(s) core–shell structures show formation of merely nanocubes. Although UV–visible spectroscopy and X-ray photoelectron spectroscopy analyses of the samples typically suggest the formation of both Ag(c)–Au(s) and Au(c)–Ag(s) bimetallic nanostructures, the definitive evidence comes from high-resolution transmission electron microscopy–high-angle annular dark field elemental mapping in the case of Au(c)–Ag(s) nanomorphs only. The resultant nanocomposite materials are used to fabricate resistors on ceramic rods having two electrodes by drop casting technique. These resistors are examined for their relative humidity (RH) response in the range (2–93% RH) and both the bimetallic nanocomposite materials offer optimized sensitivity of about 20 Kohm/% RH and 300 ohm/% RH at low and higher humidity conditions, respectively, which is better than that of individual nanoparticles.

## 1. Introduction

Bimetallic nanoparticles have emerged as an increasingly important research area, because of their interesting catalytic, electronic and optical properties which are quite distinct from those of the corresponding monometallic counterparts [1–6]. Alloyed and layered are the two major forms of bimetallic nanostructures. One of the examples in layered type is core–shell nanostructures. Several types of core–shell nanostructures, such as heterogeneous metal/metal [7–9], metal/semiconductor [10–12], metal/polymer and other combinations [13–16], have been explored hitherto. Among such systems, noble metal core–shell nanostructures have received a lot of attention due to their fascinating optical and electronic properties. In particular, Au and Ag bimetallic systems have attracted enormous attention not only because of their chemical stability, but also due to their distinctive properties in catalysis, surface-enhanced Raman scattering and biomedical applications [4,5,17–21]. Besides, nanostructures including core–shell, coupled or alloy, have a high surface-to-volume ratio and thus mass transfer and heat transfer properties are better than those of the corresponding bulk materials. Hence, nanoparticles *per se* can play an important role in the field of sensors.

Various chemical methods are now available for the synthesis of both Ag(c)–Au(s) and Au(c)–Ag(s) core–shell nanostructures. For example, Ag and Au alloy nanoparticles of certain bimetallic compositions can be synthesized when salts of both metals are simultaneously reduced [4,5,8,9]. Core–shell Ag–Au colloids generated via successive reduction of the different metal salts have also been investigated [18–20]. In the preparation of core–shell nanostructures by colloidal chemistry method, polymers are usually used as shielding agents to prevent particle aggregation. At the same time, the chemisorption of functionalized group of polymers on metal particles can lead to an efficient method for surface modification.

In the last few years, there has been continuously growing interest in humidity sensors in various fields such as air-conditioning systems, medical and industrial equipment, wherein the research is mainly focused on polymeric humidity-sensitive material [22]. Large number of research reports deal with the interaction between polymers and water vapour and evaluation of their electrical properties as a function of the relative humidity (% RH) [23–27]. However, the exact mechanism of ion transport in humidity is not known. Poly(vinyl alcohol) (PVA), being a water-soluble polymer possesses inherent characteristics such as adhesion, processability, biocompatibility and complexity. In our previous work, we have taken advantage of these properties of PVA to synthesize individual noble metal nanostructures and studied their optical properties [28,29]. We herein report a simple chemical route for the synthesis of Au(c)–Ag(s) and Ag(c)–Au(s) core–shell bimetallic nanocubes and nanoprisms in PVA matrix. These core–shell nanocomposites when tested for humidity sensing applications disclosed better sensitivity than that of the individual monometallic nanoparticles.

## 2. Experimental

### 2.1. Synthesis of bimetallic nanostructures

All the chemicals and reagents were of analytical grade. Double distilled water was used throughout the work. Aurochloric acid and silver nitrate were obtained from SD Fine Chemicals while PVA (MW 125 000) and hydrazine hydrate were purchased from Qualigens. Hydrazine hydrate was used in dilution with water. Three sets of experiments were performed for the preparation of the core–shell nanostructures. The first and second experiment sets involved preparation of Ag(c)–Au(s) and Au(c)–Ag(s) core–shell nanostructures through successive reduction of silver/gold metal salts in two steps. In the first experiment, initially, PVA (1 g) was dissolved in 25 ml of water to generate a viscous solution. Ten millilitres of 0.01 M silver salt solution was then added to this solution. The reaction mixture was thoroughly stirred at room temperature for 30 min to ensure homogeneous mixing. Dilute solution of hydrazine hydrate was separately prepared in water and 20 µl of it was added to the reaction mixture by a micro-syringe. The colour of the solution instantly changed to pale yellow. After complete reduction, the reaction mixture was further stirred for 30 min. To this reaction mixture, 10 ml of 0.01 M gold salt solution was added drop-by-drop with constant stirring which was further subjected to reduction with dilute hydrazine hydrate solution. The colour of the reaction mixture changed to violet after subsequent reduction of gold salt solution over silver nanoparticles. The second experiment was similar to the first one, except that initially gold salt solution was reduced to generate ruby red solution which was turned into orange colour after subsequent reduction of silver salt solution.

In the third experiment, mixed salt solution of silver and gold (in the same ratios used in above two experiments) was simultaneously reduced in PVA matrix using hydrazine hydrate. The colour of the reaction mixture was found to be brown. Self-standing nanocomposite films were also prepared via solution casting method by using these nanocomposite dispersions.

### 2.2. Physico-chemical characterization of the resultant nanostructures

UV–visible spectra of as-prepared solutions after the reduction of metal ions were recorded with UV spectrophotometer (Perkin Elmer) in the wavelength range of 300–800 nm. X-ray photoelectron spectroscopy (XPS) analysis of the nanocomposite films was carried out with a VG MicroTech ESCA 3000 instrument at a pressure better than 1 × 10^−9^ Torr. The general scan and C 1s, Au 4f and Ag 3d core level spectra were recorded with monochromatized Mg Ka radiation (photon energy = 1253.6 eV) at a pass energy of 50 eV and electron take-off angle (angle between electron emission direction and surface plane) of 60°. The overall resolution of measurement is thus approximately 1 eV for the XPS analysis. The core level spectra were subjected to background correction using the Shirley algorithm and the chemically distinct species were resolved using a nonlinear least-squares procedure. The surface morphology and particle size were determined with field-emission scanning electron microscopy (FESEM; Hitachi S-4800). For this purpose, the samples were prepared by mounting a drop of a nanocomposite dispersion on an alumina stub and allowing it to dry in air. In order to acquire high imaging resolution and spatial resolution for atom to atom chemical mapping of the material, we employed high-resolution transmission electron microscopy equipped with a high-angle annular dark-field detector (STEM-HAADF) and electron energy loss spectroscopy (EELS) elemental mapping using a JEOL, JEM ARM200F operated at 200 kV with spherical aberration corrector. The samples for this purpose were carefully prepared by dispersing the resultant films in de-ionized water and a drop of the dispersion was then transferred to carbon coated grid. For atomic force microscopy (AFM) imaging of Ag(c)–Au(s) and Au(c)–Ag(s) nanocomposites, the polymer films which embedded the bimetallic nanostructures were cut into 5 × 5 mm^2^ size and placed on a metal puck using instant glue. AFM images were obtained using a Multimode Nanoscope (Veeco, USA) in the air tapping mode.

### 2.3. Humidity sensing measurements

The nanocomposite materials were further used to fabricate resistors on ceramic rods having two electrodes by drop casting. The thickness of the resistor was increased with layer by layer deposition of (2 µl) solution per layer. Closed humidity system for testing the humidity responses of the films was set up in house. It consists of a glass chamber with a small neck. The chamber was kept on a glass sheet to make it air tight. Sample and the standard humidity sensors were kept in the chamber and connected to a digital multimeter for resistance measurement. RH was created by passing water vapour. When the RH meter shows 100% RH, the saturated vapours were wiped by tissue paper by gently lifting the chamber. The chamber was dehumidified by using dehumidifiers phosphorus pentoxide (P_2_O_5_), calcium carbonate or silica gel, etc.

## 3. Results and discussion

The most salient aspect of our work is the synthesis of prismatic nanostructures of Ag(c)–Au(s) core–shell with such a simple method. PVA prevents agglomeration and gives well-stabilized core–shell structures. These materials, when tested for humidity sensing, offered relative humidity response in the range (2–93% RH) and both the materials were found to exhibit optimized sensitivity of ∼20 Kohm/% RH and 300 ohm/% RH in low and higher humidity range, respectively.

### 3.1. UV–visible spectroscopy

**Figure 1.**
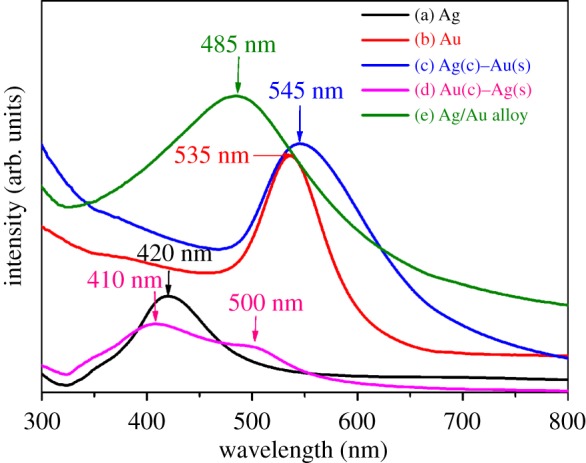
UV–visible spectra of individual nanoparticles and the bimetallic reaction products.

Figure 1 displays the optical absorption spectra of pure metal colloids (Ag and Au) and three different reaction mixtures. Pure Ag nanoparticles have characteristic surface plasmon resonance (SPR) peak at 420 nm (figure 1*a*), similar to the signal detected in a previous report [28]. After addition of HAuCl_4_ solution, the colloid exhibits an Au-like SPR peak at 545 nm (figure 1*c*) which indicates that optical properties of composite nanoparticles are dominated by gold shell. Compared to monometallic Au nanoparticles which have a peak wavelength of 535 nm (figure 1*b*), the peak wavelength of the Ag–Au nanocomposite appears to be red-shifted by 10 nm. This difference can be attributed to the core–shell morphology of the resultant nanostructure. If Ag/Au alloy nanoparticles or individual Au and Ag mixed colloids were formed, the SPR band would have exhibited a blue-shift with increasing Ag seed volume [30].

Similarly, interfacial reduction of silver ions over gold nanoparticles can result in the relative broadening and increase in absorption intensity at 410 nm (figure 1*d*) in addition to blue shifting of the longer wavelength peak of monometallic Au at 535 nm (figure 1*b*) to 500 nm (figure 1*d*). The two different absorption peaks indicate two types of collective electron oscillations in the formation of core–shell nanoparticles [31]. The enhancement in the peak intensity at 410 nm can be due to an increase in the thickness of the Ag layer on the Au seeds. The shifting of the longer wavelength band (initially around 530 nm) to 500 nm after subsequent reduction of Ag over Au could be attributed to the formation of an Au–Ag alloy at the interface of the Au core and the Ag shell [32]. Here, the bimetallic alloy formation at core–shell interface could have taken place during the initial stages of reduction process when the surface of PVA-capped Au nanoparticle is coated with few atomic layers of metallic Ag. Further reduction, however, could have led to the deposition of pure Ag on the already formed alloy interface. However, the alloy formation is confined only to the interface of core–shell and not in totality because there is an absence of single absorption band with the intermediate band position to that of individual monometallic Au and Ag nanoparticles [7]. Formation of individual Ag or Au nanoparticles during this process can also be ruled out as there is absence of two distinct peaks at 535 and 420 nm arising from the SPR of monometallic Au and Ag, respectively. From these spectral features, it is evident that there is formation of Au(c)–Ag(s) core–shell structure with reasonable indications of alloy formation at the interface of core and shell.

Furthermore, the reaction mixture of simultaneously reduced Ag and Au salt displays a single broad absorption peak at 485 nm (figure 1*e*) which is intermediate to the individual absorption peaks of monometallic Ag and Au nanoparticles observed at 420 and 535 nm, respectively. This peak presumably authenticates the formation of Ag–Au alloy type of structure.

### 3.2. X-ray photoelectron spectroscopy analysis

**Figure 2.**
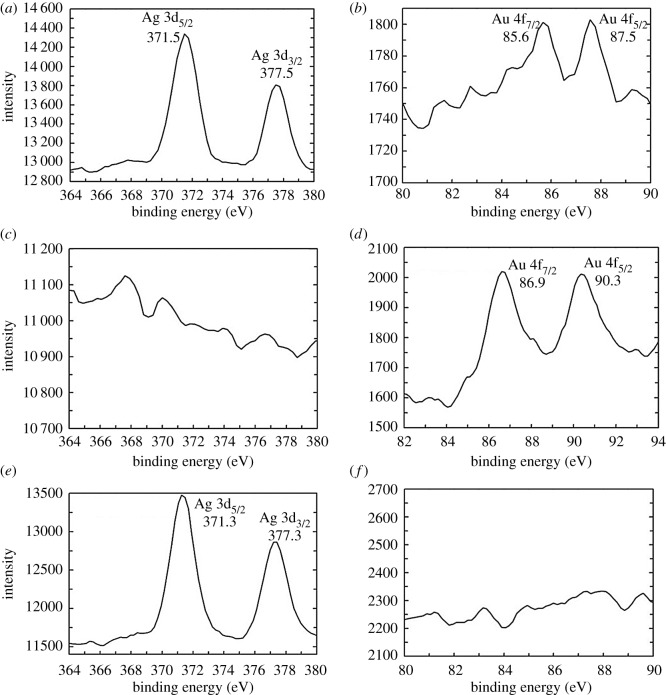
High-resolution XPS spectra of (*a*) Ag, (*b*) Au, (*c*,*d*) Ag (core)–Au (shell) nanocomposite and (*e*,*f*) Au (core)–Ag (shell) nanocomposite.

In order to support the above results, the chemical composition of core–shell nanostructures was determined using XPS. The spectra were recorded from self-supported films of the respective samples prepared by solution casting. The background correction was made using the Shirley algorithm [33] and the core levels were aligned with respect to the adventitious C 1s binding energy (BE) of 285 eV. Figure 2 presents high-resolution XPS scans of the individual and bimetallic nanostructures. Individual monometallic Ag nanocomposite showed two obvious peaks at 371.5 (Ag 3d_5/2_) and 377.5 eV (Ag 3d_3/2_) (figure 2*a*). Similarly, for monometallic Au nanocomposite, two peaks at 85.6 (Au 4f_7/2_) and 87.5 eV (Au 4f_5/2_) are observed (figure 2*b*). In the case of Ag(c)–Au(s) core–shell, emission from the Ag core level appears to be suppressed (figure 2*c*) and only the emission from Au shell prevails at 86.9 eV (Au 4f_7/2_) and 90.3 eV (Au 4f_5/2_) (figure 2*d*) which, in turn, substantiates the formation Ag (core)–Au (shell) nanostructure owing to the fact that XPS is surface sensitive technique. Although it implies the presence of Au in a single chemical form, that of its metallic state, the sizable shifts in BE values of Au 4f components can be the consequence of electronic perturbations in nanoscale Ag–Au core–shell configuration. In the case of Au(c)–Ag(s) core–shell nanostructures prepared by subsequent reduction of Ag over Au nanoparticles, the two chemically distinct Ag 3d components are observed at BEs of 371.3 and 377.3 eV (figure 2*e*). The low BE component is attributed to electron emission from silver nanoparticles (nanoshell). Relatively prominent BE signals from Ag could be detected (figure 2*e*), but there was suppression of signal arising due to Au core (figure 2*f*). This could be understandable as the amount of Au in the shell was too small to be detected which, in turn, endorses formation of Au(c)–Ag(s) nanostructure. It may be noted that in the case of probable formation of Au–Ag mixed alloy structure in lieu of Ag–Au or Au–Ag core–shell structures, we would have detected prominent signals attributable to both Au and Ag moieties in the relevant XPS spectra of our nanocomposites.

### 3.3. Morphological/microstructural examination

**Figure 3.**
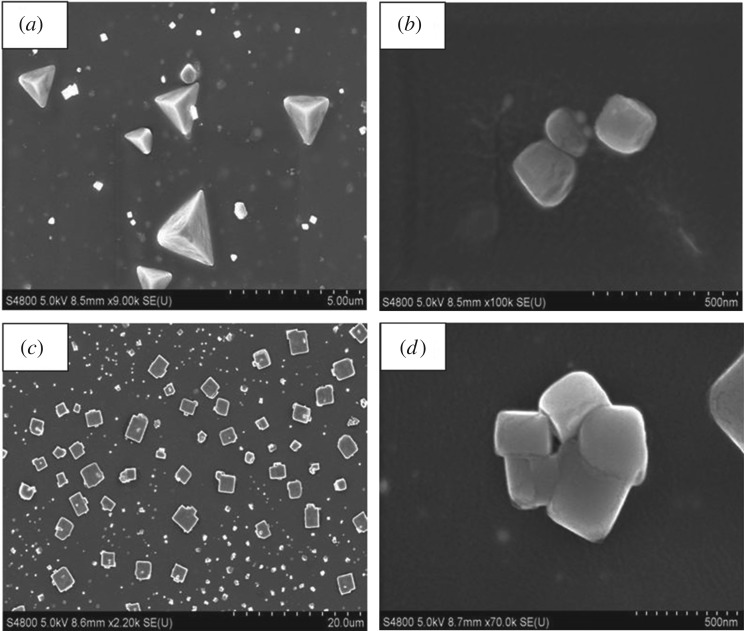
FESEM images of typical Ag (core)–Au (shell) nanocomposite as shown in (*a*,*b*) and Au (core)–Ag (shell) nanocomposite as shown in (*c*,*d*).

Typical FESEM images of the resultant core–shell nanocomposites are reproduced in figure 3. Bright shells and dark cores are clearly indicated in the images. FESEM images of Ag(c)–Au(s) nanocomposites exhibited formation of nanocubes along with nanoprismatic structures (figure 3*a,b*). It is interesting to observe that for Au(c)–Ag(s) samples, only cubical morphology is observed (figure 3*c,d*). Although the edge size of the cubes ranges from 50 to 100 nm, few larger cubes (edge size approximately 250 nm) were also observed due to fusion of the particles (figure 3*d*) The size of Ag(c)–Au(s) nanocubes is found to be less when compared to that of Au(c)–Ag(s) nanocubes. Nevertheless, the huge variation in particle size (polydispersity) is observed in the case of both the nanostructures.

**Figure 4.**
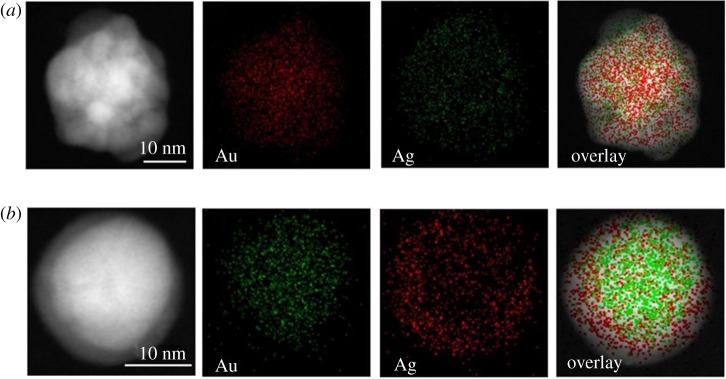
STEM-HAADF-EELS mapping images corresponding to (*a*) Ag (core)–Au (shell) and (*b*) Au (core)–Ag (shell) nanocomposites.

Owing to the similarity in the crystal structure and lattice constant, it is cumbersome task to differentiate Au and Ag regions in bimetallic nanostructures just by examination of the interplanar distances obtained in their high-resolution transmission electron microscopy images. Hence, fine-scale microstructural examination of the resultant bimetallic core–shell nanostructures in the present case was carried out by STEM-HAADF imaging and associated EELS elemental mapping. Although atomic number (*Z*)-based contrast difference offered by STEM-HAADF imaging normally allows one to locate the relative positions of the different elements, it is difficult to interpret such images in bimetallic core–shell nanostructures produced by galvanic replacement. We faced this situation in the case of STEM-HAADF image and accompanying elemental mapping data of Ag(c)Au(s) nanostructures as seen in figure 4*a*. The intermixed existence of bright and grey patches/portions of STEM-HAADF image correspond to mapping regions of Au(red colour) and Ag(green colour) at some locations only. Therefore, unequivocal chemical zonation of Ag and Au towards central (core) and surface (shell) regions could not be noted in the mapping image of Ag(c)Au(s) nanomorphs (figure 4*a*). At this juncture, it may be recalled that the pertinent UV–visible spectral feature reveals predominantly Au-like SPR peak implying Ag(c)Au(s) configuration. Besides, an absence of significant blue-shift in the SPR peak as well as preponderance of bimetallic core–shell formation in sequential reduction process weakens the possibility of bimetallic Ag–Au alloy formation. On the contrary, STEM-HAADF image of Au(c)Ag(s) nanomorph (figure 4*b*) indicates Au core as brighter central region and Ag shell as lighter (grey) halo around brighter central region. This observation is quite consistent with earlier studies [34]. The overlap of corresponding mapping images clearly reveals segregation of Au (green colour) at central region (core) and that of Ag (red colour) towards outer region (shell). Thus, the elemental mapping and STEM-HAADF image conjointly provide the conclusive evidence for the formation of Au–Ag core–shell nanomorphs only.

**Figure 5.**
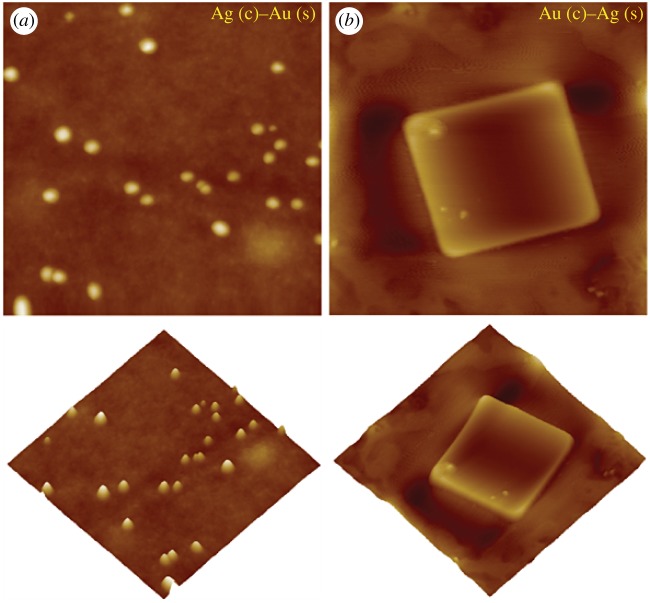
Representative two-dimensional and three-dimensional AFM images of (*a*) Ag (core)–Au (shell) and (*b*) Au (core)–Ag (shell) nanocomposites. The scan sizes of all AFM images are 1 × 1 µm^2^.

Figure 5 displays the typical two-dimensional (top) and three-dimensional (bottom) AFM images of Ag (core)–Au (shell) and Au (core)–Ag(shell) nanocomposites. The core–shell nanocomposites made by Ag(c) and Au(s) showed the proper prism like structures with a diameter of 20–25 nm. Further, core–shell nanocomposites made of Au(c) and Ag(s) showed the defined nanocube structure with a diameter of 200–250 nm. Although the AFM images of the nanocomposites were protruding structures from PVA polymeric thin films, its size and shape were well matched with the FESEM images. The AFM imaging and analysis of size and shape was more precise than other surface studies. The evolution of specific nanoscale morphologies (i.e. nanoprisms and nanocubes) in the resultant bimetallic core–shell structures can be ascribed to precise interaction between metallic species and functional groups of stabilizing polymer matrix which presumably precedes the reduction process [8] and also to competing growth rates between {111} and {100} facets of isostructural Au and Ag.

### 3.4. Humidity sensing performance

**Figure 6.**
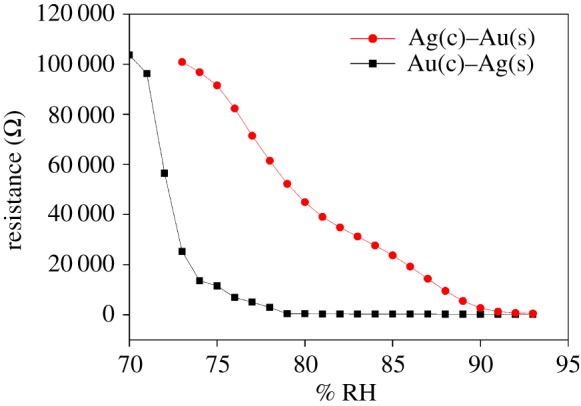
Resistance as a function of % RH for first layer of Au–Ag and Ag–Au core–shell nanostructures.

Subsequent change in the resistance as a function of relative humidity was measured for single layer of deposition of solution (shown in figure 6). The lower limit for humidity sensing exhibited by these resistors is 70 and 75% RH for Ag(c)–Au(s) and Au(c)–Ag(s) samples, respectively. Ag(c)–Au(s) changes its resistance monotonically. Two regions can be seen in the sensing behaviour. For Au(c)–Ag(s), region 1 (low humidity) is in between 73% and 90% RH with sensitivity of 6 Kohm/% RH and region 2 (high humidity) is in between 88% and 93% RH with sensitivity of 1100 ohm/% RH. (b) For Ag(c)–Au(s), region 1 (low humidity) is in between 70% and 75% RH with sensitivity of 22 Kohm/% RH, while region 2 (high humidity) is in between 76% and 93% RH with sensitivity of 2000 ohm/% RH. In both the regions, sensitivity offered by Ag(c)–Au(s) is more.

For enhancing the applicability of sensors for sensing the full humidity range, the thickness of the resistor is increased because the sample thickness affects the diffusion of water molecules inside the film through porosity and also by the absorption capacity through number of active sites of the film. From plots of resistance versus % RH (figure 6), it appears that the required thickness of Ag(c)–Au(s) sample to cover the full humidity range will be less than that of Au(c)–Ag(s) sample.

**Figure 7.**
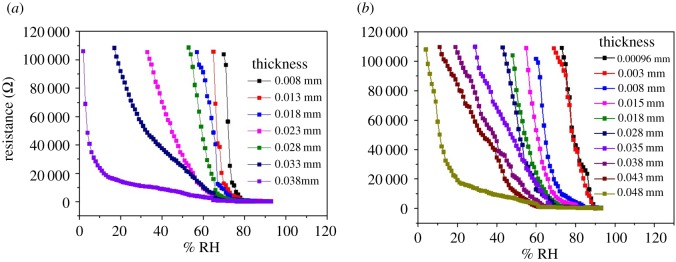
Plot of resistance as a function of relative humidity for (*a*) Ag–Au core–shell (*b*) Au–Ag core–shell nanocomposites.

The thickness dependent humidity sensing behaviour of Ag–Au and Au–Ag core–shell nanocomposites is shown in figure 7 and their comparative performance is tabulated in tables 1 and 2, respectively. The lower humidity sensing range goes on increasing with increase in thickness to cover almost the full range of humidity (3–100% RH) at thickness of 0.038 mm for Ag(c)–Au(s) and at 0.048 mm for Au(c)–Ag(s) (figure 7*a,b*). With increase in the thickness, the humidity sensing range increases exhibiting nonlinear behaviour of the two regions (lower thickness up to 0.018 mm for Ag(c)–Au(s) and 0.028 mm for Au(c)–Ag(s) samples; the thickness of the samples was measured by micrometre screw gauge having least count of 0.001 mm). Both the regions reveal increase in their range with thickness at the cost of sensitivity. At still higher thickness, both the samples continue to show nonlinear behaviour with three regions at the cost of sensitivity. From figure 7, one can conclude that at low humidity range, the sensitivity is the highest for each sample. In the mid-region, it is lower than the first region and at the highest region, it is the lowest. The tunability of low humidity sensing limit is better for Au(c)–Ag(s) samples though their sensitivity is less than those offered by Ag(c)–Au(s) samples. In the third region (low humidity), resistance decreased by about two orders of magnitude, from 10^5^ ohm to 10^3^ ohm, whereas in the second region (middle humidity), resistance decreased by about an order of magnitude, from 10^3^ ohm to 10^2^ ohm. In the first region (high humidity), gradual decrease (few tens of ohms/% RH) in the resistance was observed without any significant change in the order of magnitude.

**Table 1.** Sensitivity in terms of humidity region of Ag–Au core–shell nanocomposite films.

		sensitivity (ohm/%RH)		
		region 1	region 2	region 3
sr. no.	thickness (mm)	(high humidity)	(middle range humidity)	(low humidity)
1	0.008	2347 (93–76)	21 130 (75–70)	
2	0.013	277 (93–72)	14 803 (71–65)	
3	0.018	285 (93–69)	8603 (68–57)	
4	0.023	38 (93–76)	709 (75–66)	82 781 (65–54)
5	0.028	37 (93–74)	588 (73–60)	36 524 (59–33)
6	0.033	24 (93–70)	1083 (69–61)	19 956 (68–17)
7	0.038	28 (93–65)	286 (64–37)	1337 (36–2)

**Table 2.** Sensitivity in terms of humidity region of Au–Ag core–shell nanocomposite films.

		sensitivity ohm/(%RH)		
		region 1	region 2	region 3
sr. no.	thickness (mm)	(high humidity)	(middle range humidity)	(low humidity)
1	0.00096	1129 (93–88)	6818 (88–73)	
2	0.003	672 (93–87)	6343 (87–70)	
3	0.008	371 (93–77)	6082 (76–60)	
4	0.018	297 (93–70)	6783 (68–55)	
5	0.018	214 (93–66)	5217 (65–48)	
6	0.028	232 (93–63)	5134 (62–43)	
7	0.038	37(93–69)	2294 (68–63)	2771 (62–29)
8	0.038	32 (93–66)	950 (65–57)	2739 (56–19)
9	0.043	30 (93–63)	821 (62–51)	2538 (50–11)
10	0.048	27 (93–62)	317 (61–40)	2325 (39–2)

To study the effect of thickness on available number of sites/porosity, we have measured the weights of the samples at room temperature and at the highest (i.e. 93%) RH. Table 3 presents the difference in water content between Ag(c)–Au(s) and Au(c)–Ag(s) samples for the selected common thicknesses, which clearly implies that Ag(c)–Au(s) samples have higher water content and hence exhibit higher sensitivity. The response and recovery times as a function of thickness of the samples are shown in table 4. The response time for Ag(c)–Au(s) samples for the full range is 43 s while, for Au(c)–Ag(s) samples, it is 52 s. The recovery time for both the samples is nearly the same (approx. 5 min).

**Table 3:** Water content of Ag–Au and Au–Ag core–shell nanocomposite films.

		water content (g)	
sr. no.	thickness (mm)	Ag–Au core–shell	Au–Ag core–shell
1	0.008	0.0019503	0.001462
2	0.018	0.0019579	0.0019589
3	0.028	0.0053711	0.0042569
4	0.038	0.0063415	0.0060523

**Table 4:** Response and recovery times of Ag–Au–PVA, Au–Ag–PVA at higher humidity to room humidity.

	Ag–Au core–shell			Au–Ag core–shell		
sr. no.	thickness	recovery time (s)	response time (s)	thickness	recovery time (s)	response time (s)
1	0.008	47	5	0.0009	25	10
2	0.013	62	12	0.003	53	21
3	0.018	170	21	0.008	96	24
4	0.023	174	37	0.018	120	34
5	0.028	252	40	0.018	149	36
6	0.033	280	41	0.028	200	36
7	0.038	296	43	0.038	240	40
8				0.038	260	42
9				0.043	280	46
10				0.048	294	52

### 3.5. Nanostructures-enabled humidity-sensing mechanism

The humidity-sensing mechanism can be explained in terms of diffusion of water in samples as a function of thickness. Diffusion depends on the porosity and thickness of the sample and also on continuity of the surface water [35]. Surface water on film plays an important role in low humidity sensing while at higher humidity, along with surface water, interior water also helps in humidity sensing though capillary action. Water, being a highly polar solvent with lone-pair of electrons, is a good donor of H^+^ and electrons, which is useful in proton conduction mechanism [36]. Protons are the dominant carriers responsible for the electrical conductivity in bulk water. Based on adsorption and capillary condensation of water, protons are produced. For ionic sensing materials, if the humidity increases, the resistivity decreases and the dielectric constant increases [37,38]. When a water molecule is adsorbed on a film surface, it splits to produce protons. More protons (H^+^) are produced when the sensing material is exposed to more humidity. In the first stage of H^+^ ions production, no proton can move because, for the first monolayer, water molecule is chemically adsorbed on an activated site to form an adsorption complex. It subsequently transfers to surface hydroxyl groups. Next water molecule is bonded with two neighbouring hydroxyl groups through hydrogen bonding. Thus, there is a restriction on the top water molecule layer condensed due to the two-hydrogen bonding. In the second step, water continues to condense on the surface which forms an extra, somewhat un-ordered layer of adsorbed water on the top of the first physically adsorbed water layer.

All the humidity sensing samples exhibit decrease in resistance on increasing % RH. Initially, water vapour chemisorbs on the surface of the sensor. The electrical response will depend on the number of water molecules adsorbed on the sensor surface [39]. Water is then physically adsorbed on top of the chemisorbed layer, on top of which further physisorbed layers will be formed. The physically adsorbed multi-layered water molecules on the surface of the thin sensing film play a dominating role in the humidity-sensing mechanism [40]. It has been reported that conduction in thin films occurs through ionic carriers, while electronic conduction at low % RH is negligible [39].

In a lower RH environment, the water content is very small. As the RH increases, the water content also increases slowly. This is likely to be related to the effect of surface film thickness on the magnitude of diffusion. At low water content, the diffusion pathways become disconnected, and diffusion effectively stops. The water molecules on the solid surface can lead to slower diffusion, so diffusion of water is effectively less and simultaneously is water content. The surface water component could serve as a predominant diffusion pathway.

Experimental results reveal the critical role of surface water in controlling transport pathways. A dry porous sample was quite resistive; however, when exposed to humidity it shows measurable resistance (*R* > 110 Mohm). The slight decrease in water content caused an increase in the measured resistance. At higher humidity, the sample is totally saturated (RH > 90%) which indicates the formation of multilayers of water on the samples deposited on the ceramic rod. It has been reported that water exists in three configurations as a function of water saturation [33]. In the samples under study, these configurations are as follows: (a) region 1 (≈2–38% RH) where only one layer of water molecules is deposited, (b) region 2 (39–62% RH) where water is adsorbed onto the side walls of the pores resulting in an increase in the water content and (c) region 3, from ≈63% to 93% RH, where water content tends to saturate. In region 3, water content is found to be maximum.

The resistance of dry sample is very high. As the % RH increases (region 1), there is a sharp drop in resistance due to protonic conduction through adsorbed water on the pores and surface sites. With further increase in water content (region 2), there is further gradual decrease in resistance due to surface site occupancy of side walls of the pores which, in turn, effectively reduces the resistance of the grains. In region 3, water approaches its saturated level and resistance goes on decreasing very slowly. To study the aging effect, the samples were retested after five months and the response was found to be reproducible.

**Figure 8.**
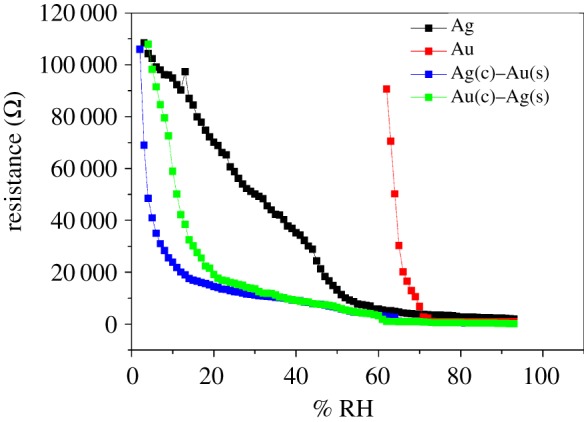
Resistance versus % RH plots corresponding to Ag, Au, Ag(core)–Au(shell) and Au(core)–Ag(shell) nanocomposites.

When the above results of core–shell nanocomposite samples were compared with those offered by individual Ag or Au nanoparticles dispersed in PVA (figure 8), it is found that bimetallic core–shell–PVA nanocomposites (both Ag(c)–Au(s) and Au(c)–Ag(s)) offer better sensitivity (2000–2500 ohm/% RH) at low humidity when compared to that of Ag–PVA and Au–PVA nanocomposites (1500 ohm/% RH). Besides, such Ag(c)–Au(s)–PVA and Au(c)–Ag(s)–PVA nanocomposites cover the entire range of humidity at thickness of about 0.038 and 0.048 mm, respectively, when compared to Ag–PVA (thickness 0.058 mm), whereas Au–PVA (thickness 0.023 mm) senses only up to 62% RH. Although such observations can be broadly linked with synergistic effect of nanoscale bimetallization in PVA matrix, we are unable to offer categorical mechanism for the same at this juncture.

## 4. Conclusion

Au(c)–Ag(s) and Ag(c)–Au(s) bimetallic nanoparticles have been successfully synthesized inside PVA matrix and further investigated for humidity sensing. The results indicated that the resultant bimetallic core–shell nanocomposite samples exhibit high sensitivity with smaller response and recovery time at low humidity compared with the individual monometallic nanocomposites. Sensitivity is also found to be persistent even after 5 months. The amount of water content in the sample was found to be enhanced with increase in the thickness of the sample.

## Data accessibility

All data relevant to this work are presented throughout the paper.
